# Examination of Replicate Syntheses of Metal Organic Frameworks as a Window into Reproducibility in Materials Chemistry

**DOI:** 10.1021/acs.jpcc.5c08003

**Published:** 2026-01-13

**Authors:** David S. Sholl

**Affiliations:** † Oak Ridge National Laboratory, Oak Ridge, Tennessee 37830, United States; ‡ Department of Chemical and Biomolecular Engineering, Rice University, Houston, Texas 77005, United States

## Abstract

Replicate experiments are a useful tool in understanding the repeatability of scientific measurements. In 2019, a systematic search for replicate syntheses of a collection of 130 metal–organic frameworks (MOFs) found that 89% of these materials had no reported replicate syntheses apart from the original publications identifying the material ( AgrawalM.,  Proc. Natl. Acad. Sci. U.S.A. 2020, 117, 877−882 10.1073/pnas.1918484117 31879338 PMC6969490). A potential weakness of that search was that only 5–11 years had elapsed since the original publication of each material. Here, this analysis is extended to all publications 11–17 years after the original publication. Although this extended time period identifies more repeat syntheses, 83% of the materials still have no reported replicate syntheses. We also consider how appropriately selected Density Functional Theory (DFT) calculations can provide corroboration for the experimentally reported crystal structures. By using data from previous high-throughput DFT studies, corroborating evidence from DFT was available for 17% of the 130 structures for which no replicate syntheses are available. In total, approximately 1/3 of the 130 MOFs have data associated with replicate synthesis experiments and/or directly corroborating DFT calculations.

## Graphical abstract

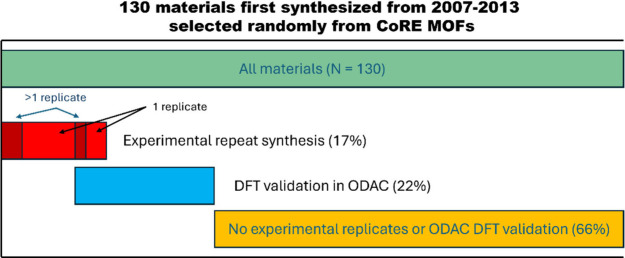

## Introduction

Understanding the reliability and replicability of data reported in the scientific literature is critical to any effort to advance a scientific field. Estimating the reliability of individual reports is a key skill developed by seasoned researchers, since basing future work on results that are uncertain (in the opinion of the researcher) is wasteful. In this mode of research it could be argued that low quality data has limited impact, assuming of course that expert researchers can correctly identify data of this type. This description does not apply, however, to the increasing number of “big data” studies that aim to collect large data sets from published reports and then make predictions using machine learning (ML) methods. In general terms these studies tend to weigh each data point equally, so low quality or erroneous data can significantly compromise ML conclusions if it is prevalent.

Because of the importance of repeatability of scientific data it is useful to move beyond anecdotal experience by systematically assessing the scientific literature. Carefully repeating experiments is perhaps the most powerful way to examine these issues. Well-publicized efforts to reproduce experimental studies in subdisciplines of biomedical science that reported low rates of repeatability^1,2^ were pivotal in drawing attention to these issues. An impressive recent analysis of reproducibility of more than 1000 claims from 52 years of literature of immunity research in *Drosophila* identified 45 claims that had not been tested in repeat experiments.^3^ A large fraction of these claims were found to be nonreproducible when tested in repeat experiments.

Systematically repeating experiments from previous studies is of course time-consuming and expensive. An alternative approach is to perform coordinated multilaboratory studies in which multiple replicates of selected experiments are used to assess the uncertainties associated with specific measurements. Examples of this approach include generation of reliable data for gas adsorption in reference materials,^4,5^ comparison of polymer characterization using gel permeation chromatography,^6^ testing methods for angle-resolved light scattering,^7^ examining reproducibility in X-ray reflectometry measurements,^8^ and analysis of systematic uncertainties in measurements of deuterium content in deuterated carbon films.^9^ This approach can also be used for computational methods, as in the work of Lejaeghere et al. comparing the performance of a range of codes for performing Density Functional Theory (DFT) calculations.^10^

Multilaboratory studies can reveal sources of significant discrepancies in the reported values of quantities that might otherwise be thought of as highly controlled. In a study involving more than 40 authors, Osterrieth et al. uncovered wide variability in data processing of N_2_ adsorption isotherm data to determine BET surface areas for porous materials.^11^ This work led to development of standardized software tools for this common analysis. In a similar example, a multilaboratory study in 1990 of measurements of extensional viscosity in dilute polymer solutions using samples prepared in a single location gave results varying by 3 orders of magnitude.^12^ This outcome spurred development of new and more reliable methods that were later demonstrated in a second multilaboratory study.^13^

Targeted replication of previous experiments and multilaboratory studies are both resource intensive. A third approach that can give information about the reliability of literature data is to search for replicates that already exist in published reports. In highly active subfields of research it is relatively common for replicate experiments to be reported because more than one investigator generates similar ideas. In a series of papers examining single-component adsorption isotherms in metal–organic frameworks (MOFs), 1062 replicate isotherms of this kind were identified.^14–16^ By applying statistical tests, 16.5% (that is, roughly 1 in 6) of these isotherms were classified as outliers relative to other replicates with the same material at the same temperature. This kind of analysis does not provide information about why some reports are outliers, but knowing that outliers exist can be a useful motivation to understand potential sources of experimental variability. In one example of this kind, Sarswat et al. performed detailed studies of the synthesis of the MOF MUF-16, determining that trace impurities in the synthesis mixture played an important role in stabilizing the adsorption properties of the resulting crystals.^17^ Identification of extant replicate experiments has also been useful in considering the reliability of mixture adsorption data in porous materials.^18^

A prerequisite for the replicate adsorption experiments described above is that the porous material of interest has been synthesized more than once. This observation suggests another tool for probing how much is known about the repeatability of literature reports for these materials, namely the likelihood that a material reported as a new material is subsequently synthesized again for any purpose. If a material is reported in the literature once but no later synthesis of the same material is known, it is not possible to draw any firm conclusion about the repeatability of the material’s properties aside from estimations based on consideration of materials that are judged to be similar.

The discussion above motivates interest in understanding how often new materials in the materials chemistry literature are synthesized after the report that describes the material for the first time. In 2019 Agrawal et al. tackled this question for metal–organic frameworks by selecting 130 MOFs that were first reported between 2007 and 2013.^19^ These materials were chosen randomly from the CoRE MOF database,^20^ a collection of thousands of experimental crystal structures. Agrawal et al. examined all papers from 2018 and earlier that cited the original papers describing each of the 130 MOFs. A key conclusion from this analysis was that 115 of the 130 MOFs (88.5%) had no reported repeat synthesis.

Agrawal et al. also emphasized that a small number of MOF structures exist that have been synthesized in many (sometimes hundreds) of separate experiments.^19^ Examples of these structures include HKUST-1 (also known as CuBTC) and ZIF-8. Agrawal et al. noted considerable variation in the reported BET surface areas of these “canonical” MOFs, an observation that in part motivated the later work on reproducibility of analysis of N_2_ adsorption isotherms by Osterrieth et al.^11^ The existence of materials that have been described in many independent reports has important implications for efforts to understand the sources of material variability.

A potential weakness of the work of Agrawal et al. is that it only considered papers relevant to the 130 randomly selected materials published up to 2018. Below, we extend the search for replicate experiments to papers published until 2024, 11–17 years after the original publications selected by Agrawal et al. We also consider if there are situations in which information from computational models, specifically, from DFT calculations, can be used to provide evidence corroborating experimentally reported crystal structures.

## Experimental Replicates of MOF Synthesis

For consistency with the work of Agrawal et al.,^19^ the same 130 MOFs used in that earlier work were considered. These 130 MOFs represent ∼2.7% of the >4700 crystal structures collected in the 2014 version of the CoRE MOF database.^20^ Each of these structures is an experimentally reported material that was first reported between 2007 and 2013. Large numbers of additional MOF structures have been reported since 2013,^21,22^ but for the purposes of this analysis it is useful to only include materials for which many years has passed since their first synthesis.

In the discussion below, repeat syntheses of the 130 MOFs between 2007 and 2018 were identified from the work by Agrawal et al.^19^ To search for repeat syntheses between 2019 and 2024, all citations of the original publication reporting each of the 130 MOFs listed by the Web of Science were collected and examined. This procedure gave 1339 publications, adding to the more than 4600 publications examined by Agrawal et al. On average, each original publication had been cited 45.8 times by 2024, with 10.3 of these citations occurring between 2019 and 2024. Of the 1339 publications examined for this work, 24.9% (334 publications) were review articles that did not contain any new data. 5.4% (72 publications) of the total were unavailable to this author and 2 publications had been retracted; these 74 publications were not considered further.

Figure 1 shows that average citations per year for each original paper using citation counts from the Web of Science. Although there is some scatter, the data for two or more years after publication is reasonably well described by the simple function *n* = 9.42 exp­(−0.164*t*), where *t* is the number of years postpublication. Assuming this simple function is valid for all *t* > 1 predicts that each publication will ultimately receive 47.7 citations on average. For each of the 14 original publications from 2007, this approach estimates that 3.5 citations will appear in the future that were not captured in the analysis presented below. Repeating this calculation for each of the publication dates for the original papers predicts that a total of 820 future citations of the set of 130 publications can be expected. Said differently, the analysis below considered 88% of all citations of the 130 original publications that are expected at any time, past or future. The original analysis by Agrawal, by contrast, covered 68% of all citations for the publications.

**Figure 1:**
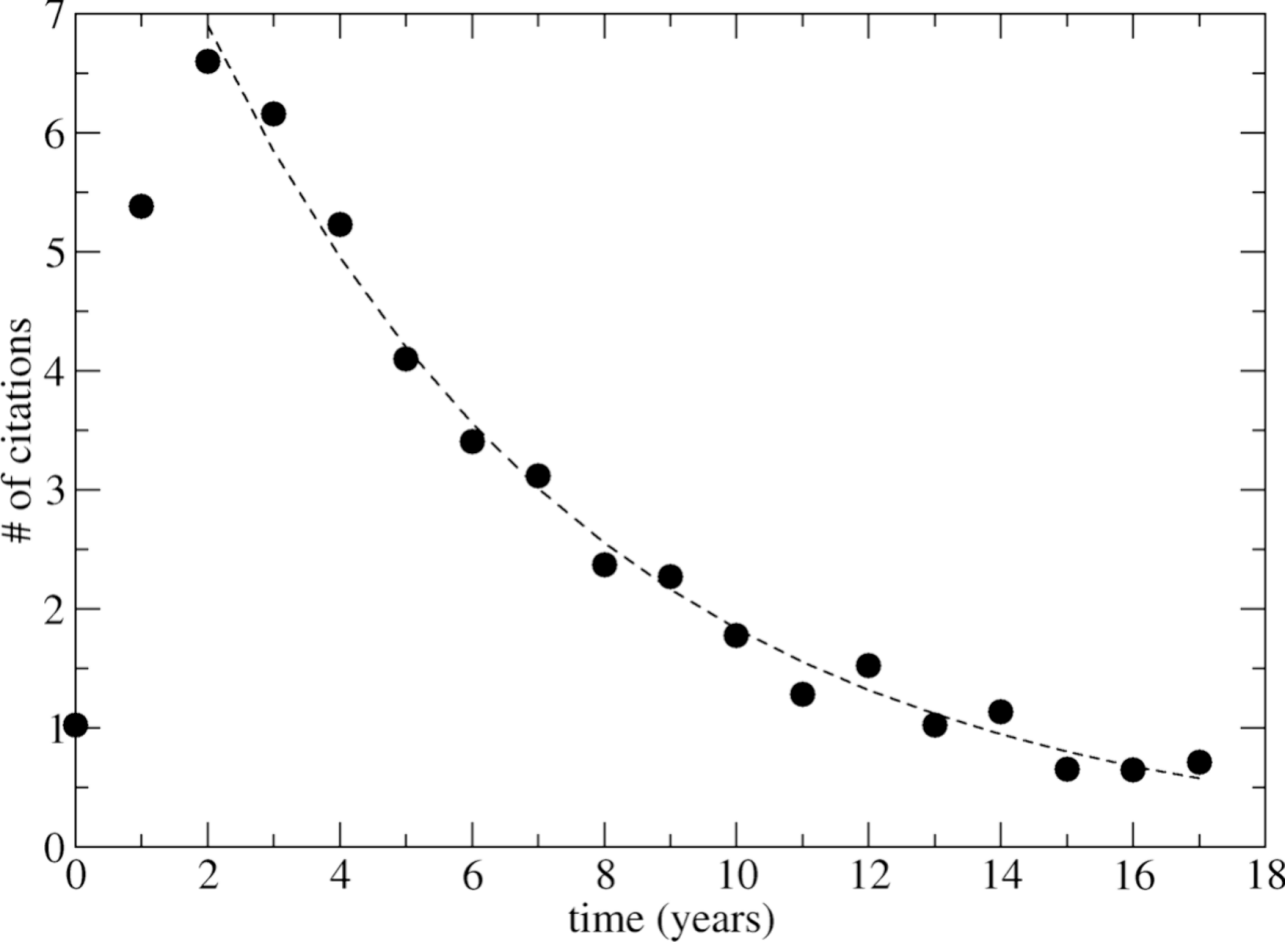
Average number of citations per year as a function of time after publication for the original papers reporting initial synthesis of the 130 selected MOFs. The dashed line is fitted to an exponential decay excluding data for 0 and 1 years.

Table 1 summarizes the number of repeat syntheses that were identified for each of the 130 MOFs considered. Extending the period of analysis to include 2019–2024 significantly increased the number of replicate syntheses reported. By 2024, repeat synthesis had been reported for 22 of the 130 materials (16.9%), 7 more materials than the period before 2019. In the period before 2019 only one material (structure code SAPBIW, also known as Bio-MOF-100)^23^ had more than two repeat syntheses. By 2024, 6 of the materials (4.6%) had more than two repeat syntheses, including structure codes IZUMUM,^24^ ZEDZAL,^25^ HOMZEP (also known as MIL-96),^26^ MACHIJ,^27^ and KEQJEX.^28^ In total, 58 repeat syntheses were reported, with SAPBIW accounting for 18 (31%) of these cases.

**Table 1:** Number of Repeat Syntheses Reported for the 130 Selected MOFs^a^

Structure code	# repeat syntheses (all years)	# repeat syntheses before 2019	# repeat syntheses 2019–2024
SAPBIW	18	6	12
IZUMUM	6	2	4
ZEDZAL	4	2	2
HOMZEP	4	0	4
MACHIJ	3	1	2
KEQJEX	3	1	2
UHISOU	2	2	0
NAYXOC	2	2	0
XUBJAF02	2	1	1
ADODAA	2	0	2
QUQGAL	1	1	0
HAWREE	1	1	0
QEGNOH	1	1	0
UFOFIF	1	1	0
MUVJIX	1	1	0
XUNGUJ	1	1	0
GEDLIM	1	1	0
TOKDON	1	0	1
XOJWEZ	1	0	1
ILITUT	1	0	1
OYUJUO	1	0	1
UVEVUN	1	0	1

a The 108 MOFs without any reported repeat syntheses are not listed.

It is interesting to ask whether repeat syntheses are evenly distributed in time. The results from 2019 to 2024 amount to 42.5% of the time since the original publications (measured in years). 59% (34 of 58) of the repeat syntheses took place during this period. If SAPBIW, which accounted for 31% of the repeat syntheses from all dates, is excluded from this analysis then 65% of the repeat syntheses occurred in 2019–2024. This data suggests that the rate at which repeat syntheses are reported per unit of time increases at dates well after the appearance of the original publication, even though the rate of new citations decreases steadily (see Figure 1).

## Quantum Chemistry Calculations as Tests of MOF Structures

The discussion above focused entirely on experimental syntheses of materials. Another potential source of information about material properties, if appropriately interpreted, is computational models. The 130 MOFs analyzed above were selected from the CoRE MOF database, a collection of structures that was generated with the specific aim of making these materials readily accessible for computational models. Although hundreds of studies have used the CoRE MOF database to simulate properties of large collections of MOFs, the majority of these studies assumed that each MOF structure was rigid in the structure reported in the CoRE MOF data set.^29^ Calculations of this kind have provided many insights into MOF properties, but they cannot be used to draw conclusions about the MOF structures themselves.

Relatively recently, large-scale Density Functional Theory (DFT) calculations have been performed for many MOFs from the CoRE MOF database and other sources. In cases where the structure of the MOFs is energy minimized without constraints on atom positions or unit cell size or shape these DFT calculations can provide a useful supplement to experimental information about MOF structure. Nazarian et al. showed by comparing DFT-optimized structures to MOFs for which high quality single crystal structures were available for solvent-free crystals that the volume of MOF unit cells from DFT is not sensitive to the DFT exchange-correlation functional and that close agreement between DFT and experiment should be expected.^30^

The ODAC23 and ODAC25 data sets of Sriram et al.^31,32^ each reported DFT-optimized MOF structures for thousands of MOFs, many of which were taken from the CoRE MOF data set. Many of the MOFs in the original CoRE MOF collection were excluded from these DFT calculations because they had very small pores or else had structural problems such as missing atoms or overlapping atoms. The challenges associated with the latter problems have largely been resolved in the most recent release of the CoRE MOF data set by Zhao et al.^22^ Other collections of DFT calculations for MOFs have also been reported, but to illustrate the concept of using this data to test MOF structures the analysis below focuses solely on data from the ODAC23 and ODAC25 data sets.

There are DFT-optimized structures in ODAC23 or ODAC25 for 50 of the 130 MOFs described above, including 39 MOFs for which no experimental repeat syntheses have been reported. A simple quantity that compares the DFT-optimized and experimental structures is the unit cell volume, *V*. There are numerous examples where the two unit cell volumes are in good agreement. The MOF with structure code TIRLIQ, for example, (*V*_exp_ = 4181 Å^3^) has *V*_DFT_/*V*_exp_ = 0.982. There are also examples, however, where the experimental and DFT unit cell volumes differ strongly, including DUQSEO (*V*_exp_ = 1305 Å^3^) with *V*_DFT_/*V*_exp_ = 0.776. In these two examples no repeat syntheses of the MOFs have been reported, so it is not possible to draw insight from experiments other than the original reported structure. Table S1 lists the unit cell volumes from DFT calculations and experiments.

Of the 50 MOFs for which DFT data was obtained, 29 of them have 0.95 < *V*_DFT_/*V*_exp_ < 1.05. For the remaining 21 cases where *V*_DFT_ and *V*_exp_ are very different it may be tempting to think that the original experimental structure is somehow faulty. This conclusion, however, is not correct. Many MOF crystal structures are determined experimentally from crystals that include ordered or disordered solvent species in the MOF’s pores. The CoRE MOF data set, however, explicitly removed solvent molecules to give the “bare” MOF, and examples are known where DFT optimization leads to pore collapse.^31^ There are also examples where the processing of experimental crystal structures to produce computation-ready structures introduced unphysical results due to missing atoms, incorrect resolution of experimentally observed disorder, and so on.^22,33^

For the reasons discussed above, it seems prudent to only consider examples with 0.95 < *V*_DFT_/*V*_exp_ < 1.05 as providing partial confirmation from DFT of the originally reported structure. This outcome certainly cannot “prove” that the reported structure is correct, and is perhaps best viewed as being similar to having two independent experiments with matching PXRD patterns. The ODAC23 and ODAC25 data sets provide confirmation of this kind for 29 of the 130 MOFs of interest, including 22 MOFs for which no repeat syntheses have been reported experimentally. Table 2 summarizes the MOFs for which repeat syntheses and/or DFT-optimized structures with 0.95 < *V*_DFT_/*V*_exp_ < 1.05 were found. It is a coincidence that the number of materials for which repeat synthesis has been reported (22/130) and the number of materials for which DFT data exists but no experimental repeats are available (22/130) are identical. It is nevertheless striking that the careful inclusion of data from computation doubles the fraction of materials for which information going beyond a single experimental report is available.

**Table 2:** MOF Structure Codes from the 130 MOFs Analyzed for Which Experimental Repeat Syntheses and/or DFT Calculations with 0.95 < *V*_DFT_/*V*_exp_ < 1.05 Were Found^a^

Experimental repeat only	Experimental repeat and DFT confirmation	DFT confirmation only
XUBJAF02 (2) TOKDON (1)	HOMZEP (4) MUVJIX (1)	HEXNII TIRLIQ
UFOFIF (1) XOJWEZ (1)	QUQGAL (1) IZUMUM (6)	KONCIA MOGNAY
UHISOU (2) ILITUT (1)	ADODAA (2) GEDLIM (1)	CUGVUW LUKLIN
MACHIJ (3) XUNGUJ (1)		MUNPAN RUCGOM
OYUJUO (1) UVEVUN (1)		CURBOH MUTVUT
KEQJEX (3) NAYXOC (2)		QUQGEP UKUBUY
QEGNOH (1) SAPBIW (18)		AXUBOL EBUREA
HAWREE (1) ZEDZAL (4)		IBUYAH NALYEG
		PAMHIW BAXSIE
		HEBKEG PEMRIK
		FEZREJ PETWOC
		SEQTEP

a Numbers in parentheses indicate the number of reported repeat syntheses. Structures are listed in the order reported by Agrawal et al.^19^

The analysis above of experimental repeat synthesis attempted to be comprehensive in the sense that it examined all published papers that cited the original publications. A similar claim cannot be made for the DFT data analyzed here, since this data was drawn from two specific sources in which DFT calculations were reported for large numbers of MOFs. It is possible that additional DFT calculations are available in the literature that would extend the list in Table 2. We reiterate that only calculations that allow the unit cell size and shape and atom positions to fully relax are useful for this purpose.

We have focused here on data from DFT calculations because unambiguous comparisons between these calculations and high-quality crystal structures have confirmed the accuracy of the calculations.^30^ Force fields of various kinds have also been used to relax large numbers of MOF structures,^34^ but the precision of these force fields is more uncertain than DFT. As general purpose force fields continue to improve,^35^ calculations with these methods could become a useful supplement to the DFT results used here.

## Discussion and Conclusion

The main aim of this study was to assess how many MOF materials have been synthesized more than once as reported in the scientific literature. Specifically, 130 MOFs were randomly selected for which 11–17 years of citation data since the original reports was available. Using 5–11 years of citation data for these MOFs, Agrawal et al. found that 89% of the materials had not been synthesized aside from their original report.^19^ Extending the analysis with 6 more years of data reduced this fraction slightly, although still 83% of the 130 materials have no reported repeat syntheses. Of the materials for which repeat syntheses have been reported, 12 (9.2%) have exactly one repeat synthesis to date and 10 (7.7%) have two or more repeats. One material (structure code SAPBIW, also known as Bio-MOF-100) accounted for 31% of all the repeat syntheses that were found.

We also explored whether some kinds of computational results could be considered as corroborating experimental syntheses. Specifically, we considered DFT calculations that allow full relaxation of the MOF crystal structure, since agreement between a calculation of this kind and experimental data can be viewed as corroboration of the experimental result. Using DFT data from previous high throughput computational studies^31,32^ we found that 29 of the 130 MOFs had 0.95 < *V*_DFT_/*V*_exp_ < 1.05. This set of materials included 22 MOFs for which no repeat syntheses have been reported experimentally. Combining the repeat syntheses and DFT corroboration, 45 of the 130 materials (35%) have some evidence of structure or properties in the literature beyond the original report. Said differently, for roughly 2 in 3 of the MOFs considered, the only direct evidence of structure or properties that is available to date is the original report. This observation has important implications for high throughput computational screening studies that aim to find “high performing” materials from large collections of known materials, namely that very little direct information about the repeatability or robustness of many of the screened materials is available.

There are multiple ways in which computational results could be used to provide actionable information that goes beyond the results listed above. In cases where DFT data is available but *V*_DFT_/*V*_exp_ < 0.95 or 1.05 < *V*_DFT_/*V*_exp_, careful comparisons with the original experimental data could be made to understand whether this discrepancy has a simple physical origin such as missing solvent species in the calculation or else could raise questions about the reliability of the original crystal structure. In cases where DFT data was not available from the specific sources we considered, targeted calculations could be performed. Similarly, other levels of theory such as force field calculations could be considered as alternative sources of information.

The question of how much information a repeat synthesis or corroborating calculation provides about the repeatability of a material’s properties is a subtle one. In many reports that include repeat synthesis the focus is on testing “new” properties or functions of a material, not quantitatively testing previously reported properties. In many examples the only reported data in common between two reports is a powder X-ray diffraction pattern. This data is suitable for qualitative identification of the material, but variations of materials with very similar diffraction patterns can have widely varying performance for properties such as gas adsorption.^17^ Nevertheless, the observation that two (or more) groups of researchers have successfully produced crystals following a stated synthesis procedure gives insight into the robustness of the material that simply cannot be known for materials that have only been reported once. Many researchers would consider evidence from experimental repeat syntheses as stronger than purely computational information. As described above, however, thoughtfully including computational data can greatly increase the number of materials for which information on any kind about repeatability is available.

It is interesting to speculate why many of the MOFs analyzed here appeared once in the literature and have not been used in further experiments. The most parsimonious explanation is simply that because the chemical space of available materials is enormous^36^ and the chemical research enterprise strongly favors originality most materials did not attract attention simply because of lack of clear motivating interests. It is also possible that some of these materials have been considered but were not used because of perceptions that they would be difficult to make or would not have “interesting” properties. There may also be cases where repeat syntheses were attempted unsuccessfully but not reported. It is not possible from the data examined in this paper to distinguish between these possibilities. Agrawal et al. found circumstantial evidence that suggested many repeat syntheses of MOFs were performed but not reported as part of studies to synthesize new variants of previous materials.^19^ The ready availability of Supporting Information in publications means that it would be helpful to the entire research community for “test” experiments of this kind to be reported even when they are not the main thrust of a new paper.

This paper focused on a very specific class of materials, MOFs, because of the availability of materials databases of crystal structures and well-defined structure codes associated with individual materials. It seems likely that the qualitative conclusion that many new chemical species or materials are not synthesized or characterized further beyond their original report also applies to many other subfields within materials chemistry. It would be interesting to perform similar quantitative analysis of experimental replicates for other classes of materials to explore how generalizable these conclusions are.

There has been considerable recent attention in the research community to the potential of using automated or self-driving laboratories to synthesize and characterize materials. We conclude with two suggestions for using facilities of this kind to advance the repeatability of synthesis of materials such as MOFs. First, automated workflows for synthesis should routinely be used to produce multiple independent samples so that data on batch-to-batch variability or consistency can be reported. This choice may not be practical during synthesis campaigns aimed at screening large numbers of potential materials, but should be routine during initial calibration of instrumentation and, just as importantly, for materials selected during screening as “interesting”. Second, a “grand challenge” problem for automated synthesis would be to systematically replicate the reported synthesis of a diverse collection of previously reported materials. To give a specific example, an effort that replicated the synthesis of the 130 MOFs analyzed above would be an extremely impressive showcase for an automated laboratory. Efforts to overcome the difficulties that would surely exist for such a showcase would undoubtedly move the field of automated synthesis forward in useful ways.

## Supplementary Material



## Notes

Tabulated data comparing experimental and DFT unit cell volumes for all MOFs considered; tabulated data underlying Figure 1 (PDF)
