# Supplementary material for: Examination of Replicate Syntheses of Metal Organic Frameworks as a Window into Reproducibility in Materials Chemistry
Source: J Phys Chem C Nanomater Interfaces. 2026 Jan 13;130(4):1433–9. doi: 10.1021/acs.jpcc.5c08003 (PMC12862813; doi:10.1021/acs.jpcc.5c08003)
Supplement: Supplementary file 1 [file jp5c08003_si_001.pdf]

Supporting Information For

Examination of Replicate Syntheses of Metal Organic Frameworks as a Window into Reproducibility in Materials Chemistry

David S. Sholl[*]

Oak Ridge National Laboratory, Oak Ridge, TN 37830, United States

*Corresponding author. Email address from January 2026: david.sholl@rice.edu

Table S1: Comparison of unit cell volumes from experimental data, V_CORE, and from fully relaxed DFT calculations, V_DFT, for structures available in the ODAC23 and ODAC25 datasets. V_CORE values were computed directly from the experimentally reported CIF files. The 130 structures are ordered using the numbering scheme defined by Agrawal et al. ODAC23 computational results are only used in cases where data for the structure is not also available in ODAC25. In the small number of cases where DFT results are from defective structures or where a choice had to be made between available structures this is noted.

PNAS #	REFCODE	# repeat synthesis	In ODAC25?	In ODAC23?	V_CORE	V_DFT	% difference	Comments
1	CETGOY		no					
2	DIXHIC		no					
3	GIQZIQ		no					
4	HEXNII		yes		847.1408	820.4847	-3.1	
5	HOMZEP	4	no	yes	5822.027	5771.526	-0.9	
6	IJOMOJ06		no					
7	KIFJIT		no					
8	PIKBUH		no					
9	RIPTAM		no					
10	SINXET		no					
11	TICPOL		no					
12	TIRLIQ		yes		4181.413	4106.153	-1.8	
13	TISGUY		no					

14	TIVYYAZ01		no					
15	XUBJAF02	2	no					
16	AFOYOK		no	yes	2508.04	2958.295	18.0	
17	COMFAM		no					
18	GOGSIF		no	yes	4552.379	4081.463	-10.3	
19	KOLWEO		no					
20	KONCIA		yes		1057.315	1057.012	0.0	
21	LOPZAS		no					
22	MOGNAY		defective	yes	2189.949	2110.804	-3.6	DFT data from ODAC23
23	NOHFOG		no					
24	RIWSUM		no					
25	SODZIV		yes		3928.154	3450.334	-12.2	
26	TOKDON	1	no	yes	1785.366	1096.333	-38.6	
27	UFOFIF	1	no	yes	1236.856	986.2002	-20.3	
28	WOCJII		no					
29	XOJWEZ	1	no	yes	784.5458	613.9631	-21.7	
30	YOMBAE		no					
31	COXFOL		no					
32	CUGVUW		defective		3743.417	3673.827	-1.9	DFT data from CUGVUW_0.08_0
33	GIYSAJ02		no					
34	GUKZAO		no					
35	LUKLIN		yes		12900.89	13421.8	4.0	
36	MUNPAN		no	yes	3813.208	3870.305	1.5	
37	OHAZAZ		no					
38	OHOLIH		no	yes	1462.328	1353.764	-7.4	
39	QUFFED		no					
40	QUTYOU		no					
41	RUCGOM		yes		3316.265	3293.897	-0.7	
42	UHISOU	2	no					
43	VACFUB01		yes		1304.906	1114.31	-14.6	

44	CESYEF01		no					
45	CURBOH		yes		8314.451	8358.056	0.5	
46	DUQSEO		yes		763.8968	592.9258	-22.4	
47	EKARUE		no					
48	GURZID		no					
49	ILITUT	1	no					
50	LUPYAX		no	yes	5210.92	4897.325	-6.0	
51	MACHIJ	3	no					
52	MUTVUT		yes		2851.186	2716.141	-4.7	
53	MUVJIX	1	yes		2231.253	2324.637	4.2	
54	NUZCER		no					
55	OSOYUR		no					
56	OWIZAW		no					
57	QUQGAL	1	yes		2507.12	2400.424	-4.3	
58	QUQGEP		yes		2583.395	2523.897	-2.3	Labeled QUQGEO_neutral in ODAC
59	RUSSAA		no					
60	RUTBUE		yes		1567.534	1023.051	-34.7	
61	RUVKOJ		yes		1290.615	874.296	-32.3	
62	UKUBUY		yes		3650.968	3655.12	0.1	
63	VAGKOF		no					
64	XUNGUJ	1	yes		827.3122	675.3888	-18.4	
65	XUYXAR		yes		2695.583	2513.731	-6.7	
66	ANEPIT		no					
67	AXUBOL		defective only		4943.633	4925.974	-0.4	DFT data from AXUBOL_0.05_0
68	EBUREA		yes		2033.35	1942.382	-4.5	
69	EMITUQ		no					
70	EPOXAJ		yes		1110.579	759.4379	-31.6	
71	EZOXEX		no					
72	HAWREE	1	yes		2681.542	1984.45	-26.0	

73	IBUYAH		yes		3662.989	3534.445	-3.5	
74	IJEXUR		no					
75	IYICUP		no					
76	IZUMUM	6	yes		2634.791	2593.395	-1.6	
77	NALYEG		yes		2851.294	2881.143	1.0	
78	OCIZIL		no					
79	OVEXOD		no					
80	OVIWIA		no					
81	OXUPUT		no	yes	3231.808	2320.596	-28.2	
82	OYUJUO	1	no					
83	PAMHIW		yes		7163.12	7078.705	-1.2	DFT data from PAMHIW_0.08_0
84	RAHNOF		no					
85	REGYOT		no					
86	UBOGAV		no					
87	UVEVUN	1	no					
88	UVINAP		no					
89	UXUYUI		no					
90	UZIJUJ		no					
91	ADODAA	2	yes		6865.583	6523.943	-5.0	DFT data from ADODAA_charged
92	BAXSIE		yes		1937.628	1963.08	1.3	
93	FAJYAS		no					
94	FAQVEA		no					
95	FATLUJ		yes		1975.965	1705.571	-13.7	
96	GEDLIM	1	yes		2744.741	2709.3	-1.3	
97	HARNAR		no					
98	HEBJAB		no					
99	HEBKEG		yes		1453.146	1423.279	-2.1	
100	HEXTAU		no					
101	KEQJEX	3	no					

102	LASMAV		no					
103	LECGIL		yes		3515.696	3222.472	-8.3	
104	MEFHUC		no					
105	NAYXOC	2	no					
106	NEFTOJ		no					
107	PEMRIK		yes		3823.231	3689.459	-3.5	
108	QEGNOH	1	no					
109	SAKNOJ		no					
110	SAPBIW	18	no					
111	SESKUY		no					
112	VEPDEB		no					
113	WIFGOJ		no					
114	ZEDZAL	4	no					
115	BETZOR		no					
116	DEYLUQ		no					
117	DEYNIG		yes		3575.977	2831.066	-20.8	DFT data from DEYNIG_0.06_0
118	FEZREJ		no	yes	4222.995	4265.971	1.0	
119	GINDEO		no					
120	LELMEW		no					
121	NIMWUD		no					
122	PETWOC		yes		2489.846	2455.343	-1.4	
123	QEWDON		no					
124	RIDGIW		no					
125	SEQTEP		yes		5055.936	4881.018	-3.5	
126	SETDUS		no					
127	SEVLEM		no					
128	VICYUD		yes		3927.963	3537.487	-9.9	
129	VIDPIJ		no					
130	ZETMOC		no					

Table S2: Numerical data plotted in Figure 1 of the manuscript.

**Yrs since pub**	**N_MOFS**	**Total cites**	**Yrs since pub**	**Avg cites**
0	130	133	0	1.023
1	130	700	1	5.385
2	130	858	2	6.600
3	130	801	3	6.162
4	130	680	4	5.231
5	130	533	5	4.100
6	130	443	6	3.408
7	130	405	7	3.115
8	130	308	8	2.369
9	130	295	9	2.269
10	130	231	10	1.777
11	130	167	11	1.285
12	114	174	12	1.526
13	89	91	13	1.022
14	66	75	14	1.136
15	43	28	15	0.651
16	31	20	16	0.645
17	14	10	17	0.714
